# The Acoustic Change Complex in Response to Frequency Changes and Its Correlation to Cochlear Implant Speech Outcomes

**DOI:** 10.3389/fnhum.2021.757254

**Published:** 2021-10-21

**Authors:** Kelli McGuire, Gabrielle M. Firestone, Nanhua Zhang, Fawen Zhang

**Affiliations:** ^1^Department of Communication Sciences and Disorders, University of Cincinnati, Cincinnati, OH, United States; ^2^Division of Biostatistics and Epidemiology, Cincinnati Children’s Hospital Medical Center, Cincinnati, OH, United States

**Keywords:** cochlear implant, hearing loss, frequency change detection, acoustic change complex, speech perception

## Abstract

One of the biggest challenges that face cochlear implant (CI) users is the highly variable hearing outcomes of implantation across patients. Since speech perception requires the detection of various dynamic changes in acoustic features (e.g., frequency, intensity, timing) in speech sounds, it is critical to examine the ability to detect the within-stimulus acoustic changes in CI users. The primary objective of this study was to examine the auditory event-related potential (ERP) evoked by the within-stimulus frequency changes (F-changes), one type of the acoustic change complex (ACC), in adult CI users, and its correlation to speech outcomes. Twenty-one adult CI users (29 individual CI ears) were tested with psychoacoustic frequency change detection tasks, speech tests including the Consonant-Nucleus-Consonant (CNC) word recognition, Arizona Biomedical Sentence Recognition in quiet and noise (AzBio-Q and AzBio-N), and the Digit-in-Noise (DIN) tests, and electroencephalographic (EEG) recordings. The stimuli for the psychoacoustic tests and EEG recordings were pure tones at three different base frequencies (0.25, 1, and 4 kHz) that contained a F-change at the midpoint of the tone. Results showed that the frequency change detection threshold (FCDT), ACC N1′ latency, and P2′ latency did not differ across frequencies (*p* > 0.05). ACC N1′-P2 amplitude was significantly larger for 0.25 kHz than for other base frequencies (*p* < 0.05). The mean N1′ latency across three base frequencies was negatively correlated with CNC word recognition (*r* = −0.40, *p* < 0.05) and CNC phoneme (*r* = −0.40, *p* < 0.05), and positively correlated with mean FCDT (*r* = 0.46, *p* < 0.05). The P2′ latency was positively correlated with DIN (*r* = 0.47, *p* < 0.05) and mean FCDT (*r* = 0.47, *p* < 0.05). There was no statistically significant correlation between N1′-P2′ amplitude and speech outcomes (all *ps* > 0.05). Results of this study indicated that variability in CI speech outcomes assessed with the CNC, AzBio-Q, and DIN tests can be partially explained (approximately 16–21%) by the variability of cortical sensory encoding of F-changes reflected by the ACC.

## Introduction

The cochlear implant (CI) is a prosthetic device that provides an effective treatment for individuals with bilateral severe-to-profound hearing loss. Currently there are approximately 750,000 CIs registered worldwide. The CI has been reported to improve hearing ability and life quality in most patients, language development in children, and possibly cognitive function in old adults (Vermeire et al., 2005; Claes et al., 2018; Andries et al., 2021).

One of the major issues about CI user’s hearing ability is that their spectral resolution is poor and they cannot perform well in tasks that heavily depend on pitch cues such as speech perception in noisy backgrounds, music melody recognition, voice pitch differentiation, and talker identification (Galvin et al., 2007; Sagi and Svirsky, 2017; Gaudrain and Başkent, 2018; Fowler et al., 2021). Unlike normal acoustic hearing with a healthy cochlea that transmits the temporal and spectral information of sounds through approximately 3,000 inner hair cells, CI users’ hearing is constrained at the peripheral stage not only by the limitation of CI signal processing algorithms that discard temporal fine structures but also by the use of only up to 22 electrodes for sound delivery (Gaudrain and Başkent, 2018; Berg et al., 2020). CI users’ capability to detect frequency changes is further exasperated by deafness (Moore, 1985). Therefore, the temporal and place cues available to CI users are limited for differentiating sound frequencies (Zeng, 2002; Pretorius and Hanekom, 2008; Swanson et al., 2009; Oxenham, 2013).

Despite similar CI technology limitations across all CI users in spectral resolution, some patients are star CI users whose performance of some tasks is within the range of normal hearing listeners’ performance while others barely benefit from their CIs (Maarefvand et al., 2013; Hay-McCutcheon et al., 2018). Understanding the source of this variability is critical for customized rehabilitation (Fu and Galvin, 2008). The variability appears to be related to a variety of potential factors including patient demographics (e.g., patient’s age, age of implantation, duration of deafness, duration of CI use, and etiology of hearing loss), cochlear abnormalities, surgical issues, electrode insertion (e.g., insertion depth and location), clinical mapping (e.g., frequency-place mismatch), device maintenance, neural status (e.g., survival of spiral ganglion neurons, and cortical neural plasticity), and higher-level cognitive functions (e.g., verbal working memory, attention, executive function, and learning processes, Blamey et al., 1992; Alexiades et al., 2001; Doucet et al., 2006; Finley et al., 2008; Reiss et al., 2008; Grasmeder et al., 2014; Jeong and Kim, 2015; Moberly et al., 2018; Berg et al., 2020; Heutink et al., 2020; Kim et al., 2021). With so many influencing factors, it is difficult to predict the likelihood of CI success using only demographic data (Lachowska et al., 2014).

Psychoacoustic methods may help us understand some of the fundamental reasons of this variability and offer single-point non-language-based measures of CI outcomes (Drennan et al., 2014). Studies have reported that CI users’ speech performance was significantly correlated to their capability to detect sound changes in spectral or frequency domain (Drennan et al., 2014; Gifford et al., 2014; Kenway et al., 2015; Sheft et al., 2015; Turgeon et al., 2015). In CI users, frequency discrimination tasks can be conducted with acoustic stimuli delivered through the sound processor, or with electrical stimuli directly presented to the CI electrodes. While the latter approach is effective to control the type of cues used (temporal vs. place cues, Nelson et al., 1995; Zeng, 2002; Kenway et al., 2015), tasks presented in the free field can provide information on patients’ performance through the sound processor, which is the way CI users perceive speech in daily lives (Martin, 2007; Pretorius and Hanekom, 2008; Vandali et al., 2015).

For frequency discrimination, many studies have used pitch discrimination or pitch ranking tasks (Gfeller et al., 2007; Won et al., 2007; Goldsworthy, 2015; Turgeon et al., 2015). These tasks require participants to identify the target stimulus that has a different pitch relative to the reference stimulus at a certain pitch or to determine the direction of the pitch change of the target stimulus relative to the reference. Therefore, these tasks assess the ability to detect across-stimulus frequency changes. Daily life sounds contain dynamic changes of acoustic features that serve as critical cues for speech and music perception. Examples of these acoustic changes include voice fundamental frequency contours, spectral shapes of the vowels, formant transitions, and melodic contours (Moore, 1985; Parikh and Loizou, 2005; McDermott and Oxenham, 2008). As described in Zhang et al. (2019), our lab used a frequency change detection task that required the participants to identify pure tones (base frequencies of 0.25, 1, and 4 kHz) containing frequency changes in the middle of the tone (within-stimulus F-changes). The results showed that there was a strong correlation (R^2^ ranges from 0.71 to 0.74) between the mean frequency change detection thresholds (FCDTs) across the three base frequencies and speech perception outcomes assessed with Consonant-Nucleus-Consonant (CNC) word, CNC phoneme, Arizona Biomedical Sentence Recognition in quiet and noise (AzBio-Q and AzBio-N), and the Digit-in-Noise (DIN) tests. Moreover, we suggested that a mean FCDT of 10% be used as a cutoff to separate CI users into moderate-to-good (e.g., >60% for CNC and AzBio-Q) and poor performers. This finding was consistent with that in Turgeon et al. (2015): proficient CI users (>65% word recognition) had a frequency discrimination threshold of less than 10% for 0.5 and 4 kHz. If the FCDT will be used as a convenient non-language test to predict CI outcomes, then it is necessary to compare CI users whose FCDT is good vs. poor to determine the neurophysiological differences of these groups. This information will deepen our understanding of neural correlates underlying patients’ variability in speech outcomes.

The auditory event-related potentials (ERPs) recorded with electroencephalographic (EEG) techniques in response to within-stimulus sound change, also called acoustic change complexes (ACCs), have attracted interests from researchers (Ostroff et al., 1998; Martin and Boothroyd, 2000; Friesen and Tremblay, 2006; Brown et al., 2015). The ACC is a type of cortical auditory evoked potential (CAEP), which could be evoked by stimulus onset (onset-CAEP), the within-stimulus sound change (ACC), and stimulus offset (offset-CAEP). The ACC does not require the individual’s attention to the stimuli or behavioral response and thus is suitable for difficult-to-test patients. The ACC measures (e.g., the minimum sound change that can evoke an ACC, the ACC peak amplitudes and latencies) were found to be in agreement with the behavioral performance of auditory discrimination tasks (Martin, 2007; Mathew et al., 2017; Han and Dimitrijevic, 2020). The ACC has shown to be reliable in normal hearing listeners, individuals with hearing loss, and CI users (Tremblay et al., 2003; Friesen and Tremblay, 2006; Martin, 2007; Martinez et al., 2013; Mathew et al., 2017).

Most previous ACC studies involving CI users have used acoustic stimuli containing changes of multiple dimensions (e.g., the changes of frequency components, intensity, and periodicity) presented in the sound field or electrical stimuli containing changes that are delivered through the CI electrode (Ostroff et al., 1998; Martin, 2007; Kim et al., 2009). Simple pure tones containing only F-changes presented in sound field can be used for both psychoacoustic and ACC experimental designs to better reveal brain-behavior relationships regarding within-stimulus detection in the frequency domain alone. Our group first examined the ACC using pure tones (160 and 1,200 Hz) containing F-changes (5 and 50%) in both normal hearing listeners (Liang et al., 2016) and CI users (Liang et al., 2018). In the CI study, we found that ACC N1′ latency to the 160 Hz tone containing the 50% F-change was significantly correlated to the behaviorally measured FCDT and clinically collected word recognition score (Liang et al., 2018). A recently published study (Vonck et al., 2021) has used tones containing F-changes to evoke the ACC in normal hearing listeners and hearing-impaired listeners. The results showed that the ACC threshold (the minimum F-change that can evoke an ACC) was significantly correlated to behavioral performance of frequency discrimination threshold. Participants with higher ACC thresholds had poorer speech perception in noise.

The current study is a companion study of Zhang et al. (2019), which did not have electrophysiological results. This study examined the ACCs evoked by F-changes at different base frequencies (f_bases_): 0.25, 1, and 4 kHz. These f_bases_ are assigned to the electrodes at different regions of the cochlea (Skinner et al., 2002). Therefore, the ACC results of the current study would provide information about how auditory cortex may process F-changes at different f_bases_ (Pratt et al., 2009).

This current study addresses the following questions: (1) How CI users process F-changes of different f_bases_ at the cortical level; (2) Can ACCs be used as objective tools to estimate the behavioral performance of F-change detection and speech perception? (3) If the mean FCDT was used to separate the CI ears into good and poor performers, as suggested in Zhang et al. (2019), what are the profiles of these two groups in demographic factors, speech performance, and ACC measures? (4) Are there within-subject ear difference between Left and Right ears in bilateral CI users? The current results would provide clinically relevant information on the substantial variability in CI outcomes across and within subjects.

## Materials and Methods

### Participants

Twenty-one adult CI users (nine females and 12 males; 20–83 years old; nine unilateral and 12 bilateral CI users) participated in this study. There was no upper age limit for subject recruitment, as previous research findings showed age is not a factor limiting CI use (Buchman et al., 1999; Wong et al., 2016). The means and standard deviations (M ± SD) of age, age at implantation, duration of deafness and duration of CI use were: 57.85 ± 14.61, 50.66 ± 16.44, 28.98 ± 18.52, and 5.22 ± 4.69 years, respectively. All participants were right-handed, native English speakers with no history of neurological or psychological disorders. All participants except two were post-lingually deafened. All CI users wore the devices from Cochlear Corporation. In the 12 bilateral CI users, eight were tested in the two CI ears separately; the rest four were tested in one CI ear only due to personal reasons for not coming back for testing in the other CI ear. Therefore, a total of 29 CI ears were tested separately. All patients have used the CI for at least 3 months (Blamey et al., 1992). The use of 3-month as a cutoff for recruitment was because: (1) Previous studies reported that CI users exhibited the greatest amount of improvement of speech perception in the first 3 months of implant use (Spivak and Waltzman, 1990; Kelsall et al., 2021). (2) One study (Drennan et al., 2014) examining the ability of adult CI users to detect spectral changes of sound over the first year of implantation reported that the improvement occurred between 1 and 3 months but not between 3 and 12 months. Demographic data of participants are shown in Table 1. This study was approved by the Institutional Review Board of the University of Cincinnati. Participants gave written informed consent before participating in the study and received financial compensation for their participation.

**TABLE 1:** Cochlear implant (CI) users’ demographics.

**Participant**	**Gender**	**Type of CI user**	**Age**	**Ear tested**	**Type of CI**	**Age at implantation**	**Duration of deafness (yr)**	**Duration of CI use (yr)**
1	F	Unilateral	61.0	L	Nucleus 6	59	25	2
2	F	Unilateral	48.0	L	Nucleus 6	45	15	3
3	F	Unilateral	66.5	L	Nucleus 6	61	47	5
4	F	Unilateral	47.4	R	Nucleus 6	45	17	2
5	M	Unilateral	59.7	R	Nucleus 6	47	20	12
6	M	Unilateral	82.9	R	Kanso	80	22	2
7	M	Unilateral	76.2	R	Nucleus 7	70	31	6
8^#^	F	Unilateral	70	R	Nucleus 7	70	72.4	2.5
9	F	Unilateral	46.0	R	Nucleus 7	44	43	1.5
10	F	Bilateral	51.4	R	Nucleus 6	42	21	9
11	F	Bilateral	20.2	L	Nucleus 6	20	18	0.25
				R	Nucleus 6	2	18	18
12	F	Bilateral	64.9	L	Hybrid^*^	64	7	0.5
				R	Hybrid	61	1	4
13	F	Bilateral	53.3	L	Nucleus 6	46	51	7
				R	Nucleus 6	38	47	15
14	F	Bilateral	54.6	L	Nucleus 6	51	46	4
				R	Nucleus 6	39	34	16
15^#^	M	Bilateral	40.9	L	Nucleus 6	39	40	2
				R	Nucleus 6	38	40	3
16	M	Bilateral	63.7	L	Nucleus 7	63	2	0.25
				R	Nucleus 6	62	2	1.5
17	M	Bilateral	64.8	L	Nucleus 6	59	9	6
				R	Nucleus 6	59	9	6
18	F	Bilateral	50.4	L	Nucleus 6	43	46	7
				R	Nucleus 6	43	46	6
19	M	Bilateral	44.5	L	Nucleus 6	41	29	3.5
20	M	Bilateral	67.7	R	Nucleus 7	63	59	4
21	M	Bilateral	78.3	L	Nucleus 7	75	23	2.5

*This participant was implanted with a Hybrid electrode array, which was clinically mapped as a standard electrode array.

*^#^Patients were prelingually deafened.*

*Duration of deafness is defined as the period from the time when hearing loss reached severe to profound level to the time of testing.*

### Stimuli

Pure tones of 1-s duration (including 20-ms raised-cosine onset and offset ramps) at f_bases_ of 0.25, 1, and 4 kHz were generated using MATLAB at a sample rate of 44.1 kHz. Then a series of tones at these three f_bases_ that contained different magnitudes of upward F-changes at 500 ms after the tone onset were generated. The F-change occurred at 0 phase (zero crossing) and there was no audible transient when the F-change occurred (Dimitrijevic et al., 2008; Pratt et al., 2009). The electrodogram of the stimuli was provided in Zhang et al. (2019), suggesting that the transient cue at the transition was minimal. The amplitudes of all stimuli were normalized.

### Procedures

Participants were first tested for pure-tone hearing thresholds to ensure the audibility of the stimuli presented through their clinical processors. They were seated on a comfortable chair in a sound-treated booth for the following tests, with the stimuli presented in the sound field at approximately 70 dBA through a speaker 1 m away from the participant’s head at 0-degree azimuth. For CI users, it is important to present the stimuli at the same perceived loudness level rather than at a fixed intensity level (Pretorius and Hanekom, 2008). Therefore, CI users were allowed to adjust their processor sensitivity setting to the most comfortable level, i.e., a loudness level of 6–7 on a 0–10 scale before testing (Hoppe et al., 2001).

#### Behavioral Tasks

##### Frequency Change Detection Threshold Test

The stimuli were tones of three f_bases_ (0.25, 1, and 4 kHz) containing F-changes, with the magnitude varied from 0.5 to 200%. The FCDT for each f_base_ was measured using an adaptive, 3-alternative forced-choice (3AFC) procedure in which the participants were instructed to identify the target stimulus by pressing the button on the computer screen with no visual feedback given. Each trial of stimuli consisted of two reference stimuli without the F-change and one target stimulus with a F-change in the middle of the tone, respectively. The order of standard and target stimuli was randomized and the silent interval between the stimuli in a trial was 500 ms. The target stimulus of the first trial was a 18% change, and the step size was adjusted according to a 2-down 1-up staircase technique based on the participants’ response. The FCDT at each f_base_ was calculated as the average of the last six reversals. Details of the FCDT were described in our previous study (Zhang et al., 2019).

##### Speech Tests

The following speech tests were administered: (1) CNC Word Recognition Test (Peterson and Lehiste, 1962). The results were scored both for words and phonemes correctly identified in terms of percent correct. (2) AzBio sentences in quiet (AzBio-Q, Spahr et al., 2012) and in noise with a signal-to-noise ratio (SNR) of +10 dB (Brant et al., 2018). Results were scored as word correctly identified in terms of percent correct. (3) Digit-in-Noise Test (DIN). Results were expressed as the speech reception threshold (SRT) in dB. A lower SRT indicates better performance (Smits et al., 2016).

#### Electroencephalographic Recordings

The 40-channel Neuroscan EEG system (Compumedics Neuroscan, Inc., Charlotte, NC, United States) was used to collect EEG data. The electrode cap was placed according to the International standard 10–20 system. Electro-ocular activity (EOG) was monitored so that eye movement artifacts could be identified and rejected during offline data analysis. The average electrode impedance was lower than 10 kΩ. EEG signals from a total of 1–3 electrodes over the CI coil were not used for recordings. During testing, participants read self-selected books or magazines to keep alert and to avoid attention effects on the ERPs. There were asked to ignore the acoustic stimuli. The continuous EEG data were recorded using tones at three f_bases_ (0.25, 1, and 4 kHz) containing different percentages of F-changes (0, 10, and 70%). There was a total of 400 trials for each of the nine types of stimuli (3 f_bases_ × 3 changes). The stimulus conditions were randomized across participants to prevent order effects. The inter-stimulus interval was 800 ms. Continuous EEG data was collected from participants with a band-pass filter setting from 0.1 to 100 Hz and a sampling rate of 1,000 Hz. The raw EEG data was saved on a computer for the following EEG data analysis.

### Electroencephalographic Data Processing

**FIGURE 1:**
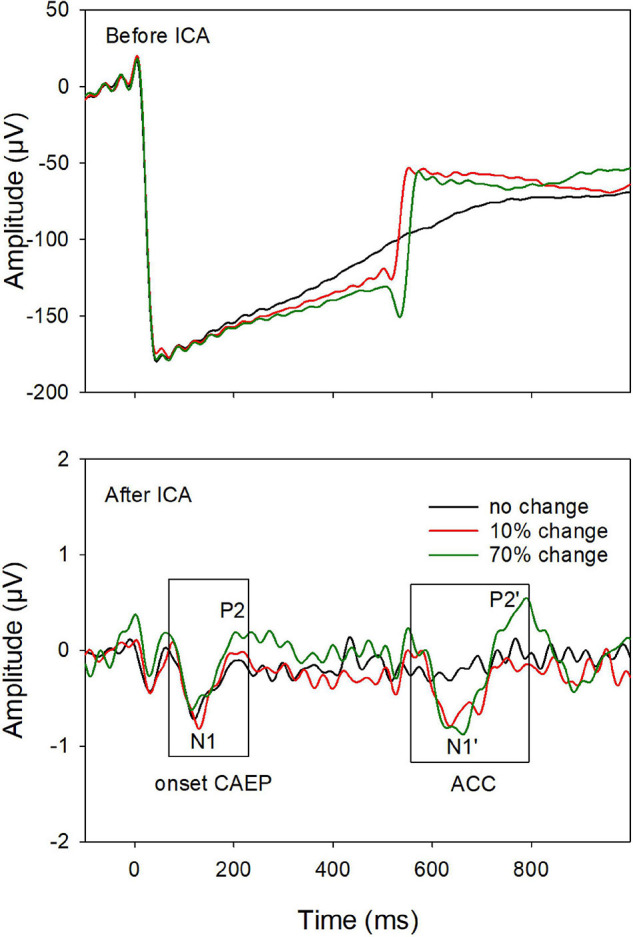
An example of event-related-potentials (ERPs) before **(top)** and after **(bottom)** artifact removal using the independent component (ICA) analysis. After removing the artifacts, the onset-CAEP and ACC are revealed. The response peaks (N1 and P2 for the onset-CAEP and the N1′ and P2′ for the ACC, as shown in the two boxes) are marked.

Continuous EEG data were digitally filtered (0.1–30 Hz) and then separated into segments over windows of −100 to 1,000 ms relative to the tone onset. Further data processing was performed using EEGLAB toolbox (Delorme and Makeig, 2004) running within MATLAB (MathWorks, United States). Each data segment was visually inspected, and segments contaminated by non-stereotyped artifacts were rejected. After this procedure, there were at least 200 segments/trials for each type of stimulus in each CI ear. The data was baseline-corrected and re-referenced using a common average reference. Independent component analysis (ICA) was then applied. The ICA decomposes the EEG dataset into mutually independent components, including those from artifactual and neutral EEG sources. The artifact components were identified using the criteria of CI artifacts (e.g., the component lasts for the whole duration of the stimulus; the scalp projection of the component shows a centroid on the CI side) and linearly subtracted from the EEG data (Gilley et al., 2006; Debener et al., 2008; Sandmann et al., 2009; Zhang et al., 2013). Figure 1 demonstrated that the ICA procedure was effective for artifact removal. Before artifact removal, large CI artifacts (more than a hundred microvolts) dominated in the EEG responses (top plot); after the artifact removal (bottom plot), the onset CAEP (N1-P2 complex), and the ACC (N1′-P2′ complex) were revealed and showed similar morphologies we reported in normal hearing listeners, despite smaller amplitudes in the CI user (Liang et al., 2016).

After artifact components were removed, the remaining components were then constructed to form the final EEG data, which was later filtered and averaged. EEG data from the electrodes close to the CI coil were replaced by linearly interpolated values computed from neighboring EEG signals. The averaged ERP waveform was derived for each type of stimuli. All ERPs were analyzed from electrode site Cz, where the cortical responses display the largest amplitude relative to other electrodes (Martin, 2007). The focus of this study is the ACC evoked by the 70% F-change, as the ACC evoked by 10% F-change was missing in multiple CI ears. The presence of the ACC was determined on the ERPs based on criteria: (i) an expected ACC wave morphology (N1′-P2′ complex) within the expected time window (approximately 580–680 ms after the tone onset), and (ii) a visual difference in the waveforms between the F-change conditions vs. no change condition. Finally, the peak components of the ACC (N1′ and P2′) were labeled. The N1′-P2′ peak-to-peak amplitude was used to represent the amplitude of the ACC, as in previous studies (Tremblay et al., 2001; Kim et al., 2009).

### Statistical Analysis

One-way repeated measure Analysis of Variance (ANOVA) was used to examine the effect of f_bases_ on ACC measures and FCDTs. Bonferroni correction was applied when assessing pairwise comparisons for f_bases_. Pearson correlation analysis was used to examine to correlation of ACC measures and other measures. All CI ears were categorized as good CI ears and poor CI ears using the 10% FCDT as a cutoff, all other data (demographic data, speech perception performance, and ACC outcomes) were compared between good and poor CI users using independent *t*-tests. All data were compared between the Left and Right ears in the eight bilateral CI users, using paired *t*-tests. Statistical significance was defined as *p* < 0.05 for all analyses. Bonferroni correction was applied to adjust the *p*-value for multiple comparisons. If data normality was not achieved and the above parametric tests were not appropriate, the corresponding non-parametric tests were used for statistical analyses. Analyses were performed in SigmaPlot Version 14 (Systat Software Inc.) and SAS Version 9.4 (SAS Institute, Cary, NC, United States).

## Results

### Acoustic Change Complex vs. Behavioral Measures

**FIGURE 2:**
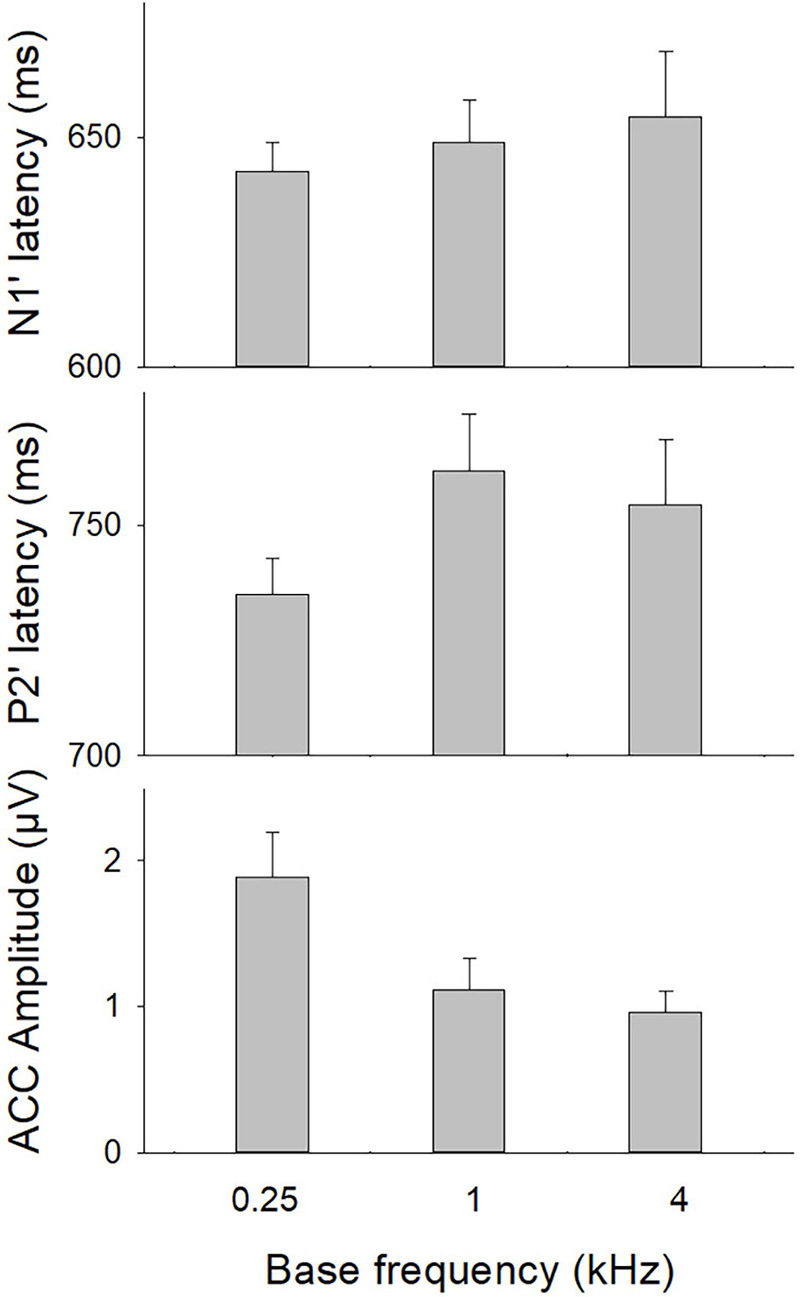
The ACC measures (N1′ and P2′ latencies, N1′-P2′ amplitude) at three base frequencies. Error bars indicate the standard error. A one-way repeated ANOVA was conducted separately for N1′ latency, P2′ latency, and N1′-P2′ amplitude. Results showed that there was a statistical significance for N1′-P2′ amplitude (*p* < 0.01). Bonferroni follow-up test showed a larger ACC amplitude for 0.25 kHz than for other frequencies (*p* < 0.01).

The ratios of present ACCs evoked by 70% F-changes, calculated with the number of present ACCs divided by the number of CI ears, were 87.5, 70.4, and 53.6% for f_bases_ at 0.25, 1, and 4 kHz, respectively. Figure 2 shows the means for the ACC measures at three f_bases_. The amplitude of the ACC was larger and peak latencies were shorter for f_base_ at 0.25 kHz than for 1 and 4 kHz.

A one-way repeated ANOVA was conducted separately for N1′ latency, P2′ latency, and N1′-P2′ amplitude. Results showed that there was a statistical significance for N1′-P2′ amplitude [*F*_(__2_,_48__)_ = 15.21; *p* < 0.01]. Bonferroni follow-up test showed that the N1′-P2′ amplitude was larger for 0.25 kHz (1.77 ± 1.42 μV) than for other frequencies (1.10 ± 0.85 μV for 1 kHz; 0.73 ± 0.57 for 4 kHz, *p* < 0.01). There was no difference in the N1′-P2′ amplitude between 1 and 4 kHz (*p* > 0.05). The N1′ and P2′ peak latencies did not differ significantly across frequencies (*p* > 0.05). Therefore, the mean N1′ latency and P2′ latency across three base frequencies were calculated and used for further correlation analyses.

The mean FCDTs at the three f_bases_ were 8.68, 4.43, and 7.69%, respectively, for 0.25, 1, and 4 kHz. One-way repeated ANOVA was conducted to examine the differences in the FCDT among different f_bases_. Normality test failed, and Friedman Repeated Measures Analysis of Variance on Ranks was conducted and the results showed no difference in the FCDT among different f_bases_ (*p* > 0.05).

The correlations between ACC measures (peak latencies and N1′-P2′ amplitude) and speech outcomes were examined using Pearson correlation analysis. The mean N1′ latency was negatively correlated with CNC word recognition (*r* = −0.40, *p* < 0.05) and CNC phoneme (*r* = −0.40, *p* < 0.05), and positively correlated with mean FCDT (*r* = 0.46, *p* < 0.05). The P2′ latency was positively correlated with DIN (*r* = 0.47, *p* < 0.05) and mean FCDT (*r* = 0.47, *p* < 0.05). There was no statistically significant correlation between N1′-P2′ amplitude and speech outcomes (all *ps* > 0.05). Table 2 shows the results of correlation analyses.

**TABLE 2:** Correlation analysis of ACC measures and behavioral performance.

	**CNC word**	**CNC phoneme**	**AzBio-Q**	**AzBio-N**	**DIN**	**Mean FCDT**
Mean ACC N1′-P2′ amplitude	–0.02	–0.06	0.12	0.11	–0.20	–0.15
	0.92	0.78	0.56	0.60	0.35	0.46
Mean ACC N1′ latency	–0.40	–0.40	–0.34	–0.21	0.40	0.50
	0.05^*^	0.05^*^	0.10	0.32	0.05	0.02^*^
Mean ACC P2′ latency	–0.30	–0.29	–0.39	–0.32	0.47	0.47
	0.13	0.16	0.05	0.11	0.02^*^	0.02^*^

*The ACC measure is significantly correlated to speech perception performance. Two *p* values of 0.05 were the result of rounding up the original *p* values.

### Profiles of “Good” vs. “Poor” Cochlear Implant Users

With the criterion of 10% FCDT, all CI ears were categorized as good CI ears (*n* = 21, mean FCDT < 10%) and poor CI ears (*n* = 8, mean FCDT ≥ 10%). Compared to poor CI ears, good CI ears showed a shorter duration of deafness (*M* = 40.38 and 26.07 years, respectively), but the difference did not reach statistical significance (*p* > 0.05). There was no difference in the duration of CI use (3.09 vs. 6.04 years, *p* > 0.05), or age at implantation (46.25 vs. 52.33, *p* > 0.05). The mean speech performance in poor CI ears was worse than in good CI ears in all tests (18.5 vs. 65% for CNC word, 34.75 vs. 77.17% for CNC phoneme, 25.9 vs. 78.2% for AzBio-Q, 16.5 vs. 54.5% for AzBio-N, and 12.5 vs. 3.9 dB for DIN). Statistical results showed that there was a significant group difference in the CNC word (*t* = −5.0, *p* < 0.05), CNC phoneme (*t* = −4.86, *p* < 0.05), AzBio-Q (Mann–Whitney *U* Statistic = 14.50, *p* < 0.05), AzBio-N (*t* = −3.65, *p* < 0.05), and DIN (*t* = 3.28, *p* < 0.05). After correcting for multiple comparisons, these statistically significant differences still existed (*p* < 0.05).

**FIGURE 3:**
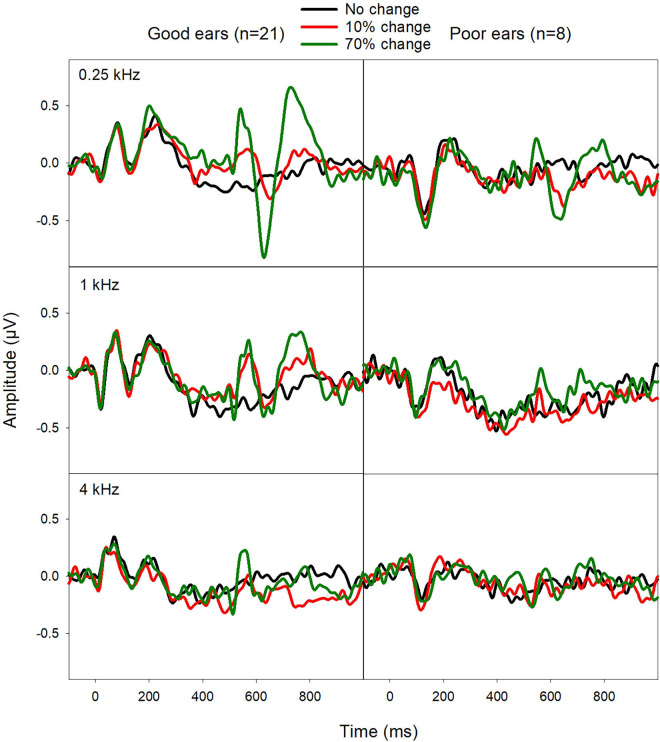
Mean ERPs from good CI ears (mean FCDT < 10%, *n* = 21) and poor CI ears (mean FCDT ≥ 10%, *n* = 8). The ACCs were more prominent for the f_base_ at 0.25 kHz than for 1 and 4 kHz. The ACCs were worse in the poor CI ears compared to the good CI ears.

Figure 3 shows the EEG data from the good and poor CI ears. Good CI ears differed from poor CI ears in the ACCs rather than the onset-CAEP. The ACC N1′-P2′ amplitude was greater for good CI ears than poor CI ears, especially for the f_base_ at 0.25 kHz. The N1′ peak for good CI users showed a shorter latency than for poor CI ears.

**FIGURE 4:**
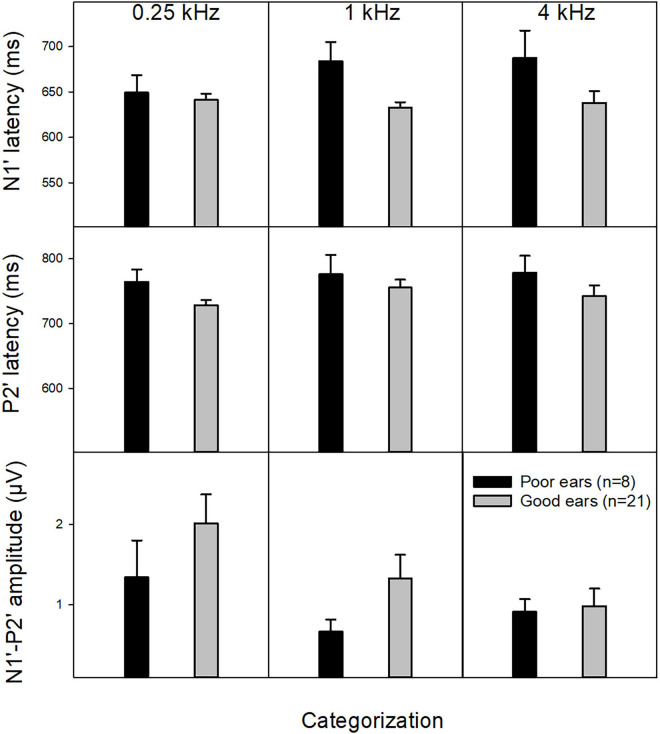
The mean ACC measures (N1′ and P2′ latencies as well as the N1′-P2′ amplitude) in good and poor CI ears at the three f_bases_. Mann–Whitney Rank Sum Test showed that the difference in ACC measures between these two subgroups did not reach statistical significance (*p* > 0.05).

The mean ACC measures evoked by 70% F-change across three f_bases_ were compared between poor and good CI ears. Mann–Whitney Rank Sum Test showed no difference in N1′-P2′ amplitude (Mann–Whitney *U* Statistic = 35.0, *p* > 0.05) N1′ latency (*t* = 1.90, *p* > 0.05), and P2′ latency (*t* = 1.77, *p* > 0.05). Figure 4 shows the mean of ACC amplitude and latencies for poor and good CI ears.

To examine if there is ear difference (within-subject variability), the data from bilateral CI users were singled out to compare between Left and Right CI ears for demographic factors (age at implantation, duration of deafness, and duration of CI use), ACC data, and behavioral data. Paired-*t* tests showed that, there was no statistical differences for age at implantation between Left and Right CI ears (48.13 vs. 42.75 years, *p* > 0.05), duration of deafness (28.50 vs. 27.25, *p* > 0.05), and duration of CI use (3.38 vs. 8.69 years, *p* > 0.05).

**FIGURE 5:**
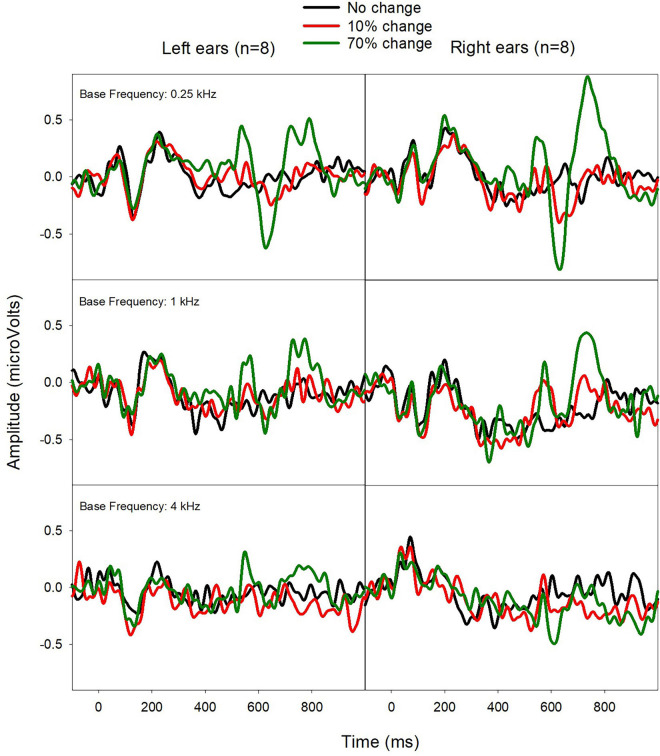
Mean ERPs from Left **(left)** and Right CI ears **(right)** in eight bilateral CI users. The ACCs were larger for Right CI ears for the f_base_ at 0.25 kHz.

Figure 5 shows the mean ERPs from Left and Right ears. The ACC shows some ear difference, with the Right CI ears displaying a larger ACC amplitude than the Left CI ears for the f_base_ at 0.25 kHz. Paired-*t*-tests showed no statistical difference in N1′ and P2′ latencies, and ACC N1′-P2′ amplitude (*p* > 0.05).

**FIGURE 6:**
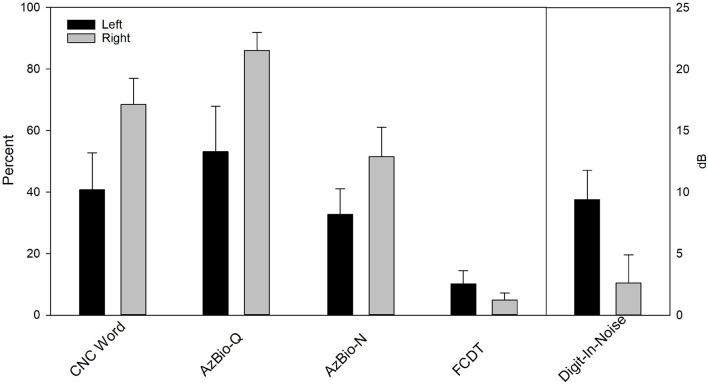
The behavioral performance for Left **(left)** and Right CI ears **(right)** in eight bilateral CI users. Wilcoxon Signed Rank Tests showed Right CI ears had better CNC score than Left CI ears (*p* < 0.05). After correcting for multiple pairs of comparisons, the difference did not reach statistical significance (*p* > 0.05).

Figure 6 shows the mean CI outcomes (speech performance and FCDT) in Left and Right ears, which suggests a better performance in Right ears. Wilcoxon Signed Rank Tests was performed and results showed Right CI ears had better CNC score than Left CI ears (*p* < 0.05), but the difference in other measures did not reach statistical significance (FCDT, AzBio-Q, AzBio-N, and DIN, *p* > 0.05). After correcting for multiple pairs of comparisons, the difference between Left and Right ears in the CNC score did not reach the statistical significance (*p* > 0.05).

## Discussion

### The Acoustic Change Complex Is a Cortical Response to Within-Stimulus Sound Changes

This study examined the ACC in response to within-stimulus F-changes and its correlation to behavioral performance of F-change detection (FCDT) and speech perception in adult CI users. The main results showed that ACC peak (N1′ and P2′) latencies were correlated with the FCDT and speech scores. Together with previous ACC studies in CI users and non-CI individuals (Martin, 2007; He et al., 2012; Kim, 2015; Liang et al., 2016; Vonck et al., 2021), our findings further support the conclusion that the ACC is a promising tool to objectively assess listeners’ ability to detect sound changes and to predict the speech perception performance.

Numerous studies have examined automatic cortical response to sound changes using another ERP, the mismatch negativity (MMN). The MMN is evoked by the oddball stimulus paradigm consisting of frequently presented standard stimuli and rarely presented deviants that differ from the standards in a certain acoustic feature (e.g., amplitude, frequency, or duration, Tervaniemi et al., 2005; Sandmann et al., 2010; Naatanen et al., 2017). However, the MMN is a neural response to the between-stimulus change (from standards to deviants that are separated with a quiet period) rather than within-stimulus sound changes, which serve as critical acoustic cues for speech perception in our daily lives. Moreover, the MMN reflects the discrepancy between the neural responses to deviants and the standard stimuli and thus it is a response to both sound change and stimulus onset rather than the acoustic change *per se*. The ACC is a true cortical response to the within-stimulus sound change and it is not affected by the stimulus onset if the duration of the sound portion prior to the occurrence of the sound change is long enough. Compared to the MMN, the ACC is more time-efficient (every stimulus trial contributes to the ACC), sensitive, and has a larger, more stable amplitude, and better test-retest reliability (Friesen and Tremblay, 2006; Kim, 2015).

### Possible Neural Mechanisms Underlying the Variability of Cochlear Implant Performance

This study has examined if the variability of CI users’ speech performance can be explained by the variability in cortical responses to within-stimulus F-changes reflected by the ACC. The variability across CI ears for each speech measure was large, with a range of 2–94% for CNC words (*M* = 52.2%), 6–98% for CNC phoneme (*M* = 65.5%), 0–99% for AzBio-Q (*M* = 65.5%), 0–95% for AzBio-N (*M* = 43.7%), and 17.7 to −5.2 dB for DIN (*M* = 6.2 dB). The mean N1′ latency was negatively correlated with CNC scores and positively correlated with the mean FCDT. The P2′ latency was negatively correlated with DIN performance and positively correlated with the mean FCDT (see Table 2). Moreover, good CI ears tend to show shorter N1′ and P2′ latencies and larger N1′-P2 amplitudes than poor CI ears (Figure 4), despite that the difference did not reach statistical significance due to small sample size in the poor CI ear group. The ACC reflects automatic sensory processing of within-stimulus sound changes, as the response was recorded without participants’ voluntary participation or attention. Therefore, the finding of the current study suggests that the variability in the cortical sensory encoding of F-changes partially (about 16–21%, *R*^2^ ≈ 0.16–0.21, *p* < 0.05) contributes to the variability in CI users’ speech perception performance.

Our current findings also indirectly indicated that, in addition to cortical sensory processing of sound, cognitive functions also play a critical role in CI speech outcomes, as suggested by behavioral studies (Moberly et al., 2017, 2018; Tamati et al., 2020). Specifically, this study found that the variability of the cortical sensory encoding of F-changes was not enough to account for the variability of speech outcomes (*R*^2^ ≈ 0.16–0.21, *p* < 0.05). In our previous study (Zhang et al., 2019), we reported that the mean FCDT across three f_base__s_ was highly correlated to the CNC, AzBio-Q, and DIN tests (*R*^2^ = 0.71–0.74, *p* < 0.05). Together, these findings may suggest that the top-down cognitive processing, which played a critical role in both behavioral performance of F-change detection and speech perception, was not reflected by the ACC that only represented the cortical sensory encoding of sound changes.

The ACC for 0.25 f_base_ displayed a larger amplitude than the ACC for other f_bases_. We suspected that this may be because the auditory pathway has uneven neural integrity for different f_bases_. Most of the participants in this study were post-lingually deafened and may have had gradual progressive deafness (most likely from high to low frequencies) before implantation. When they were fitted with hearing aids, their perception of sounds at lower frequencies was better than high frequencies even if their hearing deteriorated over time. Their prolonged low frequency hearing experience might have helped slow down the deafness-related neural degeneration along the auditory pathway. Therefore, when sounds were reintroduced after cochlear implantation, their cortical sensory encoding of F-changes (reflected by the ACC) was more robust for the low f_base_ than higher f_bases_.

It is interesting to notice that, although the ACC at 0.25 kHz was greater than that at higher f_bases_, the FCDTs at different f_bases_ were not significantly different. Several previous studies examining CI users’ frequency discrimination ability using tones presented in sound field also reported no statistical differences between low vs. high frequency ranges. For instance, Turgeon et al. (2015) reported no statistical difference in the frequency discrimination thresholds between 0.5 vs. 4 kHz (approximately 9% vs. 8% for proficient CI users and 25% and 20% for non-proficient CI users, respectively). Pretorius and Hanekom (2008) reported the frequency discrimination threshold expressed by the ratio of the actual threshold frequency to the reference frequency was constant in the tested frequency range from 200 to 1,200 Hz. Gfeller et al. (2002) reported similar frequency discrimination thresholds at 200, 400, 800, 1,600, and 3,200 Hz, with many of the tested CI users being able to discriminate frequency differences smaller than 6%. One explanation for a similar performance in the high vs. low frequency range, according to some authors, is that CI users can use non-temporal cues such as place cues alone or other cues (e.g., sound brightness related to timbre) for pitch change detection in the high frequency ranges (Swanson et al., 2009). With the ACC data available in this study, we also speculate the top-down modulation, which is not reflected by the ACC, may have played a role in behavioral performance of sound discrimination (Parker et al., 2002; Schneider et al., 2011).

### Left vs. Right Ears in Bilateral Cochlear Implant Users

Ear differences (left vs. right ears) in bilateral CI users have been reported in some studies using behavioral assessment methods (Henkin et al., 2008, 2014; Kraaijenga et al., 2018), however, neurophysiological data is lacking in the literature for adult CI users. This study reveals some ear difference in speech perception performance (CNC word, *p* < 0.05), but the significance disappeared after correcting for multiple comparisons. The ACC also shows a slightly larger amplitude for the Right CI ears relative to the Left CI ears for the low f_base_, but the difference did not reach statistical significance (*p* > 0.05). The ear difference was not shown in major demographic data (e.g., duration of CI use, age at implantation, and duration of deafness). It is likely that the small number of bilateral CI users (*n* = 8) may have prevented the potential ear difference from being revealed. However, our interesting data provide a direction for future research efforts to examine if there is a statistically significant and clinical relevant ear difference using a larger sample size.

### Clinical Implications

The FCDT test used in this study consists of pure tones with only a within-stimulus change in the frequency domain. The unique feature of this test is that it avoids interference by the acoustic changes of other domains. This study found that a 10% FCDT can separate CI users with good and poor performers, who also showed a significant difference in speech performance (*p* < 0.05). This task may provide an easy, quick, and non-linguistic tool to “screen out” poor CI ears for target intervention. This tool is useful when a clinical evaluation is not realistic or when the patients are not reliable to perform clinical speech tests (e.g., young children), or when the patients have language barriers (e.g., non-native speakers).

The capacity of ACCs in reflecting automatic auditory discrimination has been of interest among researchers looking for objective tools for assessing auditory discrimination ability. The ACC peak latencies evoked by the within-stimulus F-change were correlated to behavioral performance of F-change detection and speech perception in CI users in our previous study (Liang et al., 2016) and the current study, despite that different f_bases_ have been used in these two studies. The ACC amplitude appeared to be a less sensitive measure than the N1′ and P2′ latencies to predict speech performance and FCDTs, although it tends to be smaller in poor CI ears than in good CI ears (Figure 4). Together with previous studies (Kim, 2015; Han and Dimitrijevic, 2020), this study further confirms that the ACC can be used as an objective tool to assess CI users’ ability to detect sound changes and to predict their speech outcomes.

### Limitations and Future Studies

This study used pure tones containing a F-change that occurred at 0 phase to evoke the ACC. There was no audible transient click reported by the participants. However, these stimuli did contain a small degree of transient cue at the transition when the change occurred, as shown in the electrodogram in Zhang et al. (2019). The spread of excitation may have introduced a loudness cues that can enhance the ACC evoked by the F-change. Moreover, because frequency discrimination performance can be affected by the position of the f_base_ relative to the frequency response of filters of the CI user’s clinical map (Pretorius and Hanekom, 2008), our fixed f_bases_ selected for testing (0.25, 1, and 4 kHz) for all participants did not take the individual clinical map differences into consideration. We will address the above issues with the following approaches: (1) We will add a transition period of a fast-logarithmic frequency modulation sweep with a frequency change [f_base_ × (F-change)] between the two segments of the tone {f_base_ and [f_base_ + (F-change)]} to prevent transient signals, as suggested in a recent study using tones containing F-changes to evoke the ACC in normal hearing and hearing impaired listeners (Vonck et al., 2021); (2) We will compare the ACCs evoked by the F-change with vs. without a simultaneous intensity change to determine the extent of contribution of the sound intensity change to the ACCs evoked by the F-change; (3) We will use individualized f_base__s_ for each CI user according to the frequency response of filters of the participant’s clinical map.

Additionally, this study did not have an age-matched control group with normal hearing, which made it difficult to assess the extent of influence from patients’ age on the ACC results. Furthermore, some heterogeneity within the CI users may result in sampling effects on the results. Future studies will include an age-matched control group and a group of homogeneous CI users.

## Conclusion

This study examined the ACC evoked by within-stimulus F-changes in adult CI users, behavioral performance of frequency change detection, and speech perception performance. The mean N1′ and P2′ latencies were significantly correlated to the mean FCDT and speech scores. Using the criterion of 10% for the mean FCDT allows the separation of good and poor CI ears with significantly different speech outcomes. The ACC amplitude was significantly larger for 0.25 kHz than for higher f_base__s_, indicating that the cortical sensory processing is more robust at 0.25 kHz. The lack of effects of f_base_ on the FCDT may be the result of an additional role of the higher-level top-down modulation in frequency change detection.

## Data Availability Statement

The raw data supporting the conclusions of this article will be made available by the authors, without undue reservation.

## Ethics Statement

The studies involving human participants were reviewed and approved by the Institutional Review Board of the University of Cincinnati. The patients/participants provided their written informed consent to participate in this study.

## Author Contributions

KM collected and analyzed the data and wrote the first draft of the manuscript. FZ designed the framework of this study, helped with data analysis, and manuscript writing. NZ helped with statistical analysis and data interpretation. GF helped with participant recruitment and data collection. All authors contributed to the article and approved the submitted version.

## Conflict of Interest

The authors declare that the research was conducted in the absence of any commercial or financial relationships that could be construed as a potential conflict of interest.

## Publisher’s Note

All claims expressed in this article are solely those of the authors and do not necessarily represent those of their affiliated organizations, or those of the publisher, the editors and the reviewers. Any product that may be evaluated in this article, or claim that may be made by its manufacturer, is not guaranteed or endorsed by the publisher.
